# Interest in Quitting e-Cigarettes Among Adult e-Cigarette Users With and Without Cigarette Smoking History

**DOI:** 10.1001/jamanetworkopen.2021.4146

**Published:** 2021-04-02

**Authors:** Amanda M. Palmer, Tracy T. Smith, Georges J. Nahhas, Alana M. Rojewski, Brandon T. Sanford, Matthew J. Carpenter, Benjamin A. Toll

**Affiliations:** 1 Department of Public Health Sciences, Medical University of South Carolina, Charleston; 2 Department of Pulmonary, Critical Care, Allergy, and Sleep Medicine, Medical University of South Carolina, Charleston; 3 Department of Psychiatry and Behavioral Sciences, Medical University of South Carolina, Charleston; 4 Hollings Cancer Center, Medical University of South Carolina, Charleston

## Abstract

This survey study compares the level of interest in quitting e-cigarettes among adults who have a cigarette smoking history vs those who do not.

## Introduction

Approximately 2.8% to 3.2% of US adults are current e-cigarette users, with a majority being current cigarette smokers or former cigarette smokers.^1^ The most common use for e-cigarettes is to quit smoking, but e-cigarette use may continue even after discontinuation of combustible cigarettes.^2^ Furthermore, those who initiate e-cigarettes to quit smoking may not be successful, leading to dual use of both tobacco cigarettes and e-cigarettes, which increases potential health harms.^3^

Previous studies have shown that people who use e-cigarettes, also called vaping, are interested in quitting.^4,5^ No published randomized clinical trials for e-cigarette discontinuation exist, and evidence on how to aid e-cigarette users in stopping is limited. It is important to understand interest in quitting among e-cigarette users, including dual users. The purpose of this study was to provide the most up-to-date estimate of interest in e-cigarette discontinuation among US adults.

## Methods

Data were collected as a part of the Population Assessment of Tobacco and Health (PATH) Wave 4 adult cohort (December 2016 – January 2018), a comprehensive, publicly available longitudinal US survey approved by the Westat institutional review board.^6^ Participants provided written informed consent. This survey study follows the American Association for Public Opinion Research (AAPOR) reporting guideline.

Outcomes of interest included: (1) those who attempted to quit e-cigarettes in the past 12 months, (2) those who plan to eventually quit e-cigarettes, and (3) level of interest in quitting (measured by a scale of 1 to 10, with higher scores indicating a higher interest in quitting). Those who endorsed a quit attempt were asked how many attempts had been made in the past 12 months. Balanced repeated replication weighting procedures were used for analyses, completed on SAS version 9.4 (SAS Institute) from October to November 2020. χ^2^ tests examined differences in prevalence for dichotomous outcomes, and *t* tests examined continuous outcomes with a 2-tailed significance threshold of *P* < .05.

## Results

Of the 30 191 adults who completed the survey, 1988 (6.5%) were identified as established e-cigarette users (every day or some days for more than 30 days; Table 1). Among the analyzed sample of 1988 e-cigarette users, 1332 (59%) were male, 1384 (75%) were non-Hispanic White, 1189 (59%) had income less than $50 000 (representing lower levels of median income and educational attainment), and most were aged 44 years or younger (aged 18-24 years: 128 participants [17.9%; 95% CI, 14.8%-21.0%]; aged 25-34 years: 75 participants [14.1%; 95% CI, 9.7%-18.5%]; aged 35-44 years: 42 participants [12.3%; 95% CI, 8.4%-16.2%]). Of the established e-cigarette users, dual users were identified as concurrent established cigarette smokers who smoked every day or some days for more than 30 days (1053 participants [53.56%]); former cigarette smokers, as those who had quit smoking more than 30 days ago (540 participants [31.0%]); and never cigarette smokers, as those who denied smoking (371 participants [14.5%]). Within the full sample, 302 participants (15.2%) endorsed having made a past-year quit attempt and 1208 participants (60.7%) endorsed future plans to quit e-cigarettes.

**Table 1.** Past-Year Quit Attempts and Plans to Quit e-Cigarettes Among Established e-Cigarette Users

Participant characteristic	Analyzed sample		Past-year quit attempt			Plan to quit		
	No.^a^	Weighted %	No.^a^	Weighted % (95% CI)^b^	*P* value	No.^a^	Weighted % (95% CI)^b^	*P* value
Overall	1988	NA	302	15%^c^	NA	1208	60.7%^c^	NA
Gender								
Male	1132	59	171	13.6 (11.3-15.9)	.71	676	59.4 (55.4-63.5)	.23
Female	856	41	131	14.2 (11.9-16.4)		532	62.5 (58.9-66.1)	
Age, y								
18-24	768	25	128	17.9 (14.8-21)	.03	444	58.3 (54.1-62.6)	.04
25-34	486	28	75	14.1 (9.7-18.5)		309	65.2 (59.8-70.5)	
35-44	289	18	42	12.3 (8.4-16.2)		195	64 (58.3-69.6)	
45-54	219	14	35	15.3 (10.2-20.4)		126	55 (45.7-64.2)	
55-64	155	10	16	6.5 (2.9-10.1)		102	63.2 (27.1-46.5)	
≥65	71	5	6	8.4 (−0.6-17.4)		32	46.5 (31.8-61.2)	
Education								
Less than bachelor	1761	88	267	14.1 (12.2-16)	.35	1059	60 (56.9-63.2)	.11
Bachelor or beyond	215	12	33	11.7 (7.3-16.1)		144	66.8 (59.3-74.2)	
Race/ethnicity								
Other^d^	574	24	112	20.6 (15.8-25.3)	<.001	338	60.1 (54.7-65.5)	.79
Non-Hispanic White	1384	75	182	11.5 (9.9-13.2)		854	61 (57.4-64.6)	
Income								
Less than $50 000	1189	59	212	17 (14.5-19.4)	<.001	715	58.9 (55.2-62.7)	.03
$50 000 or more	676	36	73	8.7 (6.7-10.7)		432	64.6 (60.5-68.8)	
e-Cigarette user type								
Dual user	1053	54	177	15.3 (12.8-17.8)	<.001	624	59 (55.2-62.8)	.02
Former cigarette smoker	540	31	52	7.9 (5.2-10.7)		357	66.1 (60.4-71.8)	
Never cigarette smoker	371	15	69	20.9 (15.2-26.6)		213	55.4 (49.2-61.6)	

Abbreviation: NA, not applicable.

^a^ Unweighted No. Raw numbers and percentages may not add up to full sample due to missing data.

^b^ Percentage responding affirmatively compared with those responding no, don’t know, refused, or missing.

^c^ Unweighted percentage.

^d^ The other category included Black, Asian, and other races, such as multiracial.

Dual users, former smokers, and never smokers differed in rates of attempting to quit in the past 12 months and plans to quit in the future. Never smokers endorsed the highest rates of past quit attempts (69 never smokers [20.9%]; 95% CI, 15.2%-26.6% vs 177 dual users [15.3%]; 95% CI, 12.8%-17.8% vs 52 former cigarette smokers [7.9%]; 95% CI, 5.2%-10.7%; *P* < .001), whereas former smokers represented the highest proportion of participants planning to quit (357 former cigarette smokers [66.1%]; 95% CI, 60.4%-71.8% vs 624 dual users [59.0%]; 95% CI, 55.2%-62.8% vs 213 never cigarette smokers [55.4%]; 95% CI, 49.2%-61.6%; *P* = .02). Although the differences were not statistically significant, former cigarette smokers rated higher interest in quitting compared with other groups (former cigarette smokers’ mean [95% CI] level of interest in quitting: 4.2 [3.9-4.4] vs dual users: 3.8 [3.6-4.0]; *P* = .06; and vs never cigarette smokers: 3.8 [3.4-4.1]; *P* = .09) (Table 2). Of those who tried to quit, no group differences were found regarding number of quit attempts.

**Table 2.** Level of Interest in Quitting and Number of Quit Attempts Among Quit Attempters by Smoking Status

	Analyzed sample^a^		Dual users^a^		Former cigarette smokers^a^		Never cigarette smokers^a^		*P* value		
	No.	Mean (95% CI)	No.	Mean (95% CI)	No.	Mean (95% CI)	No.	Mean (95% CI)	Dual users vs Former cigarette smokers	Dual users vs never cigarette smokers	Former cigarette smokers vs Never cigarette smokers
Level of interest in quitting (1-10)^b^	1968	3.9 (3.8-4.1)	1053	3.8 (3.6-4)	539	4.2 (3.9-4.4)	368	3.8 (3.4-4.1)	.06	.79	.09
No. of past-year quit attempts^b^	301	2.4 (1.9-2.8)	176	2.1 (1.6-2.7)	52	2.5 (1.6-3.3)	69	3 (1.9-4)	.52	.18	.47

^a^ Unweighted No. The raw numbers and percentages may not add up to full sample due to missing data.

^b^ Level of interest in quitting was asked of the full sample, whereas number of quit attempts was only asked to those who had endorsed trying to quit in the past year. When analyzed adjusting for significant demographic characteristics from Table 1 (age, race/ethnicity, income), results were similar to the unadjusted results listed here in Table 2.

## Discussion

A majority of e-cigarette users expressed interest in eventually quitting vaping. Not surprisingly, since e-cigarette use is often initiated to quit cigarettes, former cigarette smokers had the highest levels of intention to quit and interest in quitting vaping. Results suggest that dual users and never smokers are somewhat less inclined to consider stopping e-cigarettes, which may be related to health and/or risk perceptions of vaping. These groups also endorsed higher rates of failed attempts to quit vaping, suggesting difficulties in stopping use. A limitation of the present analysis is that the anticipated timeframe for discontinuing e-cigarettes is unknown.

There is an urgent need for development of interventions to help individuals quit vaping, regardless of their cigarette smoking status. Future research should also continue to monitor characteristics of e-cigarette users interested in quitting.
